# Are Adults With Bipolar Disorder at Increased Cardiovascular Risk due to Their Physical, Biochemical, and Physiological Profiles? The FINEXT‐BD Study

**DOI:** 10.1002/brb3.70297

**Published:** 2025-02-09

**Authors:** José Etxaniz‐Oses, Sara Maldonado‐Martín, Inaki Zorrilla, Ilargi Gorostegi‐Anduaga, Maria J. Apodaca‐Arrizabalaga, Ana González‐Pinto

**Affiliations:** ^1^ GIzartea, Kirola eta Ariketa Fisikoa Ikerkuntza Taldea (GIKAFIT), Society, Sports, and Exercise Research Group, Department of Physical Education and Sport, Faculty of Education and Sport‐Physical Activity and Sport Sciences Section University of the Basque Country (UPV/EHU), Lasarte kalea, 71 Vitoria‐Gasteiz, Araba/Álava Basque Country Spain; ^2^ Bioaraba Health Research Institute, Physical Activity Exercise, and Health Research Group Vitoria‐Gasteiz Basque Country Spain; ^3^ Bioaraba Health Research Institute Mental Health and Childhood Research Group Vitoria‐Gasteiz Basque Country Spain; ^4^ Osakidetza Basque Health Service, Psychiatry Department Araba University Hospital Vitoria‐Gasteiz Basque Country Spain; ^5^ Department of Neurosciences University of the Basque Country UPV/EHU Vitoria‐Gasteiz Basque Country Spain; ^6^ CIBER of Mental Health (CIBERSAM) Institute of Health Carlos III Madrid Spain; ^7^ Bioaraba Health Research Institute, Research and Innovation in Cardiovascular Disease Group Vitoria‐Gasteiz Basque Country Spain; ^8^ Osakidetza Basque Health Service, Cardiology Department Araba University Hospital Vitoria‐Gasteiz Basque Country Spain

**Keywords:** bipolar disorder, cardiorespiratory fitness, cardiovascular risk factors

## Abstract

**Introduction:**

Bipolar disorder (BD) is associated with considerable morbidity and premature mortality, mainly due to somatic causes. This study aims to determine some physical, exercise capacity–related physiological variables and biochemical markers of health status in adults (45.4 ± 13.1 years) with BD (*n* = 65) compared to a healthy control (HC) population (*n* = 29) sample and to estimate cardiovascular risk (CVR) through different methods in the BD group.

**Methods:**

Multiple assessments included body composition, cardiorespiratory fitness (CRF), and biochemical parameters. CVR was calculated using the Framingham Heart Study, SCORE2, and relative risk methods.

**Results:**

The BD population, compared to the HC, showed unfavorable body composition (waist‐to‐hip ratio, 0.9 ± 0.1 vs. 0.8 ± 0.1; fat body mass, 33.3 ± 10.2 vs. 24.3 ± 8.9%, *p* ≤ 0.001), CRF (peak oxygen uptake, 25.2 ± 8.2 vs. 33.4 ± 8.7 mL kg^−1^ min^−1^; and cardiorespiratory optimal point, 27.9 ± 4.2 vs. 23.6 ± 4.2 ventilation/oxygen uptake ratio, *p* ≤ 0.05), biochemical concentrations of atherogenic indexes (total cholesterol/high‐density lipoprotein cholesterol ratio, 4.1 ± 1.5 vs. 3.3 ± 1.0; and triglycerides/high‐density lipoprotein cholesterol ratio, 2.8 ± 2.3 vs. 1.5 ± 1.0, *p* ≤ 0.05), and inflammatory C‐reactive protein (3.8 ± 10.2 vs. 0.9 ± 1.05 mg/dL, *p* ≤ 0.05). Consequently, CVR showed higher values (*p* ≤ 0.05) in BD (high risk, 3.1%) compared to HC (low‐to‐moderate risk, 2.2%) participants, according to SCORE2, higher (*p* ≤ 0.05) vascular age (49.8 years) than chronological age (45.8 years), with a significant difference (*p* = 0.005) compared to HC.

**Conclusions:**

This study highlights the importance of specific physical, biochemical, and physiological screening and CVR and vascular age assessment for people with BD. The practical application of these findings would prevent cardiovascular disease in BD and promote a healthier lifestyle as an adjuvant strategy to pharmacological intervention.

## Graphical abstract

Importance of specific physical, biochemical, and physiological screening and CVR and vascular age assessment for people with BD is highlighted. The practical application of these findings would prevent cardiovascular disease in BD and promote a healthier lifestyle as an adjuvant strategy to pharmacological intervention.

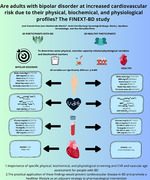

## 1 Introduction

Bipolar disorder (BD) is a mood disorder (manic and depressive symptoms), and it is associated with significant morbidity and mortality (Radua et al. 2023). In European countries, the lifetime prevalence of BD is estimated to range from 1% to 6%, with little evidence of a gender difference and a reduced life expectancy of 11–20 years compared with the general population (Kessing et al. 2021). In those people with BD aged 15–44 years, external causes of death (suicides in particular) contribute more to premature mortality than somatic causes; in older ages (i.e., 45–64 years), however, external and somatic causes contribute nearly equal numbers to excess mortality (Paljärvi et al. 2023). In this sense, alcohol‐related etiologies and infectious, respiratory, and cardiovascular diseases (CVD) contribute to the largest groups of deaths due to somatic causes (Biazus et al. 2023; Paljärvi et al. 2023). It seems that there is a vascular–bipolar link since early diagnosis in youth that accelerates atherosclerosis and CVD in adulthood (Goldstein et al. 2020, 2015, 2020, 2015). This process is due not only to the direct effects of the disease itself, including pathophysiological factors (i.e., inflammation, oxidative stress, autonomic and endothelial dysfunction), but also to indirect effects related to behavioral and environmental factors (i.e., sleep disruption, smoking, and suboptimal nutritional pattern and physical activity) (Goldstein et al. 2020, 2015, 2020, 2015).

Considering the above, the guidelines of the European Psychiatric Association for several mental illnesses to improve the care of patients recommend the assessment of CVD risk by score‐risk charts, including a relative risk chart based on smoking habits, systolic blood pressure, and total cholesterol (TC) (De Hert et al. 2009). However, current cardiovascular risk (CVR) prediction tools do not include cardiorespiratory fitness (CRF), knowing that a low CRF is associated with all‐cause mortality and CVD morbidity and mortality (Harber et al. 2017; Kaminsky et al. 2019). In this sense, cardiopulmonary exercise testing (CPET) is considered the gold‐standard assessment of CRF with peak oxygen uptake (VO_2peak_) as the maximal variable and cardiorespiratory optimal point (COP; the lowest minute ventilation to oxygen consumption ratio) as the submaximal one (Peterman et al. 2022). Both variables reflect the optimal interaction between the respiratory and cardiovascular systems, with the VO_2peak_ being the maximum amount of oxygen that can be absorbed, transported to, and utilized by working tissues during dynamically strenuous exercise, considering higher values as better (Kaminsky et al. 2019), and the COP as the minimum ventilatory equivalent for oxygen (VE/VO_2_) at any given minute during an incremental exercise test, with lower values considered better (Peterman et al. 2022). Unfortunately, despite the accumulating evidence on their clinical utility as established risk factors for CVD (Kaminsky et al. 2019; Laukkanen et al. 2021), both variables are not routinely measured, and much less in populations with mental disorders.

Therefore, this study aims to: 1) determine some physical, exercise capacity–related physiological variables and biochemical markers of health status in adults with BD compared to a HC population sample and 2) estimate CVR through different methods in the BD group.

## 2 Methods

The design, inclusion and exclusion criteria, and procedures for the FINEXT‐BD study were detailed previously (García et al. 2020). All participants gave informed consent after verbal and written information about the study.

### 2.1 Study Participants

The FINEXT‐BD study was conducted between October 2019 and June 2023 in Vitoria‐Gasteiz (Basque Country, Spain). In the baseline study, 65 participants (FINEXT‐BD) aged between 18 and 65 years (mean 45.4 ± 13.1 years), 28 men (43.1%), and 37 women (56.9%) were included. The sample of HCs (*n* = 29, 43.4 ± 12.4 years, 55.1% men and 44.9% women) was recruited from the community, with exclusion criteria encompassing any chronic medical conditions, regular intake of prescription medications, current medical symptoms, abnormal findings during physical examinations (e.g., blood pressure readings ≥ 140/90 mmHg), or abnormal outcomes on the screening tests, both at rest and during exercise electrocardiograms (García et al. 2020).

All FINEXT‐BD participants were diagnosed with BD in compliance with the *Diagnostic and Statistical Manual of Mental Disorders*, Fifth Edition (DSM‐V) (American Psychiatric Association 2013).

### 2.2 Measurements

Body Mass Index (BMI), dividing the total body mass by the square of the height (kg m^−^^2^), and waist and hip circumferences (cm), to determine the waist‐to‐hip ratio (WHR), were assessed in all participants. Furthermore, using bioelectrical impedance (Tanita, BF 350, Arlington Heights, Illinois, USA), additional metrics such as fat‐free mass (FFM) and fat body mass (FBM) were derived.

A Lode Excalibur Sport Cycle Ergometer from Groningen, The Netherlands, was used to perform Symptom‐limited CPET, starting at 40 W for the BD group and 70 W for HCs, incrementing by 10 W per minute following a ramp protocol. To determine peak oxygen uptake (V̇O_2peak_), an expired gas analyzer with a calibrated system (Ergo CardMedi‐soft S.S., Belgium Ref. USM001 V1.0) was used (García et al. 2020). Blood pressure was monitored before, during, and after the exercise test to ensure ongoing assessment of the participants’ physiological responses. Expired gas was analyzed with a system (Ergo CardMedi‐soft S.S., Belgium Ref. USM001 V1.0) calibrated before each test for the determination of VO_2peak_, which was defined as the highest value of oxygen consumption reached toward the test's end. The attainment of the VO_2peak_ was assumed from the presence of two or more of the following: 1) volitional fatigue (> 18 on the Borg scale), 2) peak respiratory exchange ratio ≥ 1.1, 3) fulfillment of > 85% of age‐predicted maximum heart rate, and 4) oxygen consumption (VO_2_) and not an increase of heart rate with increasing work rate (García et al. 2020). The accomplishment of COP was considered at the nadir of the ventilation ratio (VE) (L min) and VO_2_ (mL kg^1^ min^1^) obtained at every minute during the maximum exercise test—the so‐called ventilatory equivalent for VO_2_ (VE/VO_2_) (Peterman et al. 2022).

Staff from the hospital collected venous blood samples (10 mL) between 8.00 and 9.00 a.m. after overnight fasting in polypropylene EDTA‐containing tubes. A fasting blood glucose level of ≥126 mg/dL characterized diabetes mellitus (DM). Additionally, the following measurements were assayed: hemoglobin, hematocrit, TC, high‐density lipoprotein cholesterol (HDL‐C), low‐density lipoprotein cholesterol (LDL‐C), and triglycerides (TGs) (García et al. 2020).

Resting blood pressure was assessed using a digital blood pressure monitor (Omron M3). Participants remained comfortably seated with their backs supported and arms free for at least 5 min before the monitor was placed around the left upper arm and activated to measure blood pressure. Age and smoking status were self‐reported. The participant's physician confirmed all medications.

The CVR profile was calculated using quantitative methods, including the Framingham Heart Study (FHS) and SCORE2. The former considered age, HDL‐C, TC, systolic pressure, DM, and smoking status risk factors. Each variable received a weighted score, and the sum of the scores for each variable was converted into a percentage representing the risk of experiencing a cardiovascular event within 10 years. Thus, a score of 10% meant there was a 10% probability of experiencing a cardiovascular event in the next 10 years. A percentage of less than 6% is considered low risk, between 6% and 20% is regarded as medium risk, and a score of at least 20% is regarded as high risk (D´Agostino et al. 2008). Conversely, SCORE2, which stratifies risk based on European population data for adults (40–69 years), involves charts considering age, sex, smoking habit, systolic blood pressure, and non‐HDL‐C (SCORE2 Working Group and ESC Cardiovascular Risk Collaboration, 2021). The categories for risk assessment for people under 50 years old are < 2.5% as low‐to‐moderate risk, 2.5% to < 7.5% as high risk, and ≥ 7.5% as very high risk (SCORE2 Working Group and ESC Cardiovascular Risk Collaboration, 2021). Additionally, the relative risk chart, incorporated in the European Guidelines for CVD prevention in people with several mental disorders, is based on smoking habits, systolic blood pressure, and TC, with the risk assessment of < 1% as low risk, ≥ 1% and < 5% as low‐to‐moderate risk, 5–10% as high risk, and ≥ 10% as very high risk (De Hert et al. 2009).

The FHS method was utilized to determine the vascular age (VA) of all participants, representing the biological age of the vascular system. It reflects the age a person with the same calculated CVR would have if all their risk factors were within normal ranges (D´Agostino et al. 2008).

### 2.3 Statistical Analysis

All variables were shown with descriptive statistics as means ± standard deviations (SDs). Independent samples *t*‐tests were used to detect differences in the parametric variables between groups. A one‐factor ANOVA analysis was used to observe significant differences among groups in different parametric variables. Cohen's *d* was calculated to describe the standardized mean difference in between‐group effect sizes. Small (*d* = 0.2), medium (*d* = 0.5), and large (*d* = 0.8) were the values to interpret effect sizes. *p* < 0.05 was set as statistical significance. SPSS version 22.0 (IBM Corp., Armonk, New York) was used for statistical analyses.

## 3 Results

Related to body composition, FINEXT‐BD participants showed higher values in body mass (mean difference = 8.694, 95% CI = 1.602–15.786%, *p* = 0.005), BMI (mean difference = 5.116, 95% CI = 2.498–7.733%, *p* ≤ 0.001), waist (mean difference = 2.723, 95% CI = 8.294–19.122%, *p* ≤ 0.001) and hip (mean difference = 6.243, 95% CI = 2.484–10.002%, *p* ≤ 0.001) circumferences, WHR (mean difference = 0.079, 95% CI = 0.038–0.121%, *p* ≤ 0.001), and FBM (mean difference = 9.038, 95% CI = 4.671–13.406%, *p* ≤ 0.001) compared to HCs. Thus, values considered CVR factors (i.e., BMI > 25 kg m^2^ as overweight, FBM = 28.8% as obesity, and waist circumference = 97.5 cm as high metabolic risk) were present in the BD group. Conversely, HCs showed values in the normal range. Both groups showed optimal blood pressure values (i.e., systolic blood pressure ≤ 120 mmHg, diastolic blood pressure ≤ 80 mmHg), with no differences (*p* > 0.05) between groups.

Regarding the biochemical profile (Table 1), FINEXT‐BD participants showed higher values above the optimal concentration of atherogenic indexes (i.e., TC/HDL‐C > 3.5; TG/HDL‐C > 2) and C‐reactive protein (CRP), a proinflammatory biomarker considered cardiometabolically unhealthy per values > 3 mg/L. The other analyzed parameters showed optimal concentration values. However, when comparing both groups, significantly higher concentrations of glucose (mean difference = 8.307, 95% CI = 2.191%–14.444%, *p* ≤ 0.05), TC/HDL‐C ratio (mean difference = 0.831, 95% CI = 0.288%–1.387%, *p* ≤ 0.005), TGs (mean difference = 43.48, 95% CI = 11.981%–74.979%, *p* ≤ 0.05), CRP (mean difference = 2.8991, 95% CI = −1.142% to 6.940%, *p* ≤ 0.05), TG/HDL‐C ratio (mean difference = 1.312, 95% CI = 0.622%–2.003%, *p* ≤ 0.05) levels were observed in the FINEXT‐BD group compared to the HCs. Conversely, lower values of HDL‐C (mean difference = −9.7, 95% CI = −17.302% to 2.097%, *p* = 0.013) in the BD group were observed. Also, we found a difference in the percentage of cigarette smoking (∆ = 80.3%, *p* ≤ 0.001), with the BD population showing increased tobacco consumption.

**TABLE 1:** Clinical characteristics of the studied populations.

Variables	FINEXT‐BD *n* = 65	HC *n* = 29	*p*_FINEXT vs. HC_	Cohen's *d*
Age (years)	45.4 ± 13.1	43.3 ± 12.4	0.478	0.16
Body mass (kg)	81.7 ± 17.8	73.1 ± 10.9	0.005**	0.58
BMI (kg m^−2^)	29.1 ± 6.1	23.9 ± 5.4	≤ 0.001***	0.9
Waist (cm)	97.7 ± 16.7	84.1 ± 9.4	≤ 0.001***	1
Hip (cm)	106.1 ± 10.9	99.8 ± 7.1	≤ 0.001***	0.68
WHR	0.9 ± 0.1	0.8 ± 0.1	≤ 0.001***	1.16
FBM (%)	33.3 ± 10.2	24.3 ± 8.9	≤ 0.001***	0.94
Resting SBP (mmHg)	118.2 ± 13.1	123.7 ± 14.7	0.074	0.39
Resting DBP (mmHg)	79.5 ± 10.9	79.4 ± 9.8	0.986	0.01
Glucose (mg/dL)	93.5 ± 17.6	85.1 ± 10.9	0.009**	0.57
TC (mg/dL)	197.9 ± 39.5	193.9 ± 31.5	0.643	0.11
HDL‐C (mg/dL)	52.2 ± 19.4	61.9 ± 14.9	0.013*	0.56
LDL‐C (mg/dL)	123.4 ± 41.3	115.1 ± 27.9	0.273	0.24
TC/HDL‐C ratio	4.1 ± 1.5	3.3 ± 1.0	0.003**	1.07
TG (mg/dL)	127.3 ± 77.5	83.8 ± 40.7	0.007**	0.49
TG/ HDL‐C ratio	2.8 ± 2.3	1.5 ± 1.0	0.005 **	0.8
Uric Acid (mg/dL)	5.7 ± 4.1	5.1 ± 1.2	0.89	0.2
CRP (mg/dL)	3.8 ± 10.2	0.9 ± 1.05	0.05*	0.4
Cigarette smoking (%)	52.3	10.3	≤ 0.001***	
Diabetes mellitus (%)	7.7	0	0.127	
MEDICATION				
Mood stabilizer (%)	65.6			
Antipsychotic (%)	38.8			
Atypical antipsychotic (%)	56.7			
Antiepileptic (%)	46.2			
Antidepressant (%)	41.8			
Benzodiazepine (%)	40.2			
Hypnotic (%)	25.3			

*Note*: Significant difference between FINEXT‐BD and HC was set at *p* < 0.05.

Abbreviations: BMI, body mass index; CRP, C‐reactive protein; DBP, diastolic blood pressure; FBM, fat body mass; HC, healthy control; HDL‐C, high‐density lipoprotein cholesterol; LDL‐C, low‐density lipoprotein cholesterol; SBP, systolic blood pressure; TC, total cholesterol; TG, triglycerides; WHR, waist‐to‐hip ratio.

*p < 0.05.

**p < 0.01.

***p < 0.001.

When the exercise capacity–related variables were assessed (Table 2), CRF was markedly lower in the FINEXT‐BD group compared to HCs according to VO_2peak_ (mL kg^−1^ min^−1^, *p* ≤ 0.001, mean difference = −8.21, 95% CI = −11.946% to 4.481%, L min, *p* = 0.006, mean difference = −0.42, 95% CI = −0.72% to 0.12%), MET_peak_ (*p* ≤ 0.001, mean difference = −2.338, 95% CI = −3.407% to 1.268%), VT_1_ (mL kg^−1^ min^−1^, *p* ≤ 0.001, mean difference = −9.831, 95% CI = −12.854% to 6.209%), COP (*p* ≤ 0.001, mean difference = −3.31, 95% CI = −1.384% to 5.236%), peak workload (*p* = 0.008, mean difference = −36.75, 95% CI = −63.74% to 9.76%), the distance covered during the test (*p* = 0.008, mean difference = −0.935, 95% CI = −1.615% to 0.255%) and peak heart rate (*p* = 0.002, mean difference = −14.01, 95% CI = −22.896% to 5.123%).

**TABLE 2:** Participants’ exercise capacity–related variables.

Variables	FINEXT *n* = 65	HC *n* = 29	*p*_FINEXT vs. HC_	Cohen's *d*
Workload_peak_ (W)	152.2 ± 80.1	188.9 ± 49.7	≤ 0.008**	0.55
Time (min)	12 ± 5.1	13.7 ± 5.2	0.152	0.33
Distance (km)	2.2 ± 1.4	3.2 ± 1.6	0.014*	0.67
SBP_peak_ (mmHg)	192.9 ± 32	192.9 ± 30.2	1	0
DBP_peak_ (mmHg)	90.3 ± 22.7	86.1 ± 14.6	0.295	0.22
HR_peak_ (bpm)	154.3 ± 20.6	168.3 ± 18.5	0.002**	0.72
VO_2peak_ (L min^−1^)	2 ± 0.6	2.4 ± 0.6	0.006**	0.57
VO_2peak_ (mL kg^−1^ min^−1^)	25.2 ± 8.2	33.4 ± 8.7	≤ 0.001***	0.97
RER_peak_	1.06 ± 0.2	1.05 ± 0.1	0.559	0.02
MET_peak_	7.1 ± 2.3	9.5 ± 2.4	≤ 0.001***	1.02
VT_1_ (mL kg^−1^ min^−1^)	15.3 ± 4.8	25.2 ± 7.3	≤ 0.001***	1.6
COP (VE/VO_2_)	27.9 ± 4.2	23.6 ± 4.2	≤ 0.001***	0.21

*Note*: Significant difference between FINEXT and HC was set at *p* < 0.05.

Abbreviations: COP, cardiorespiratory optimal point; DBP_peak_, peak diastolic blood pressure; HC, healthy control; HR_peak_, peak heart rate; MET_peak_, peak metabolic equivalent of task; RER_peak_, peak respiratory exchange ratio; SBP_peak_, peak systolic blood pressure; VE/VO_2_, lowest minute ventilation to oxygen consumption ratio; VO_2peak_, peak oxygen uptake; VT_1_, first ventilatory threshold.

*p < 0.05.

**p < 0.01.

***p < 0.001.

**FIGURE 1:**
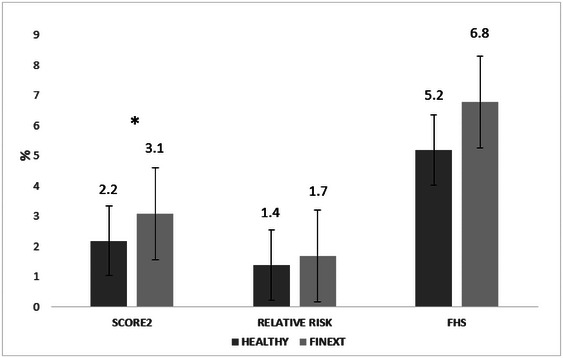
Comparison of cardiovascular risk estimation in the studied groups. FHS: Framingham heart study; RELATIVE, relative risk; SCORE, systematic coronary risk estimation. ***p < 0.001; **p < 0.01; *p <0.05: significant difference between FINEXT‐BD and healthy control (HC).

**FIGURE 2:**
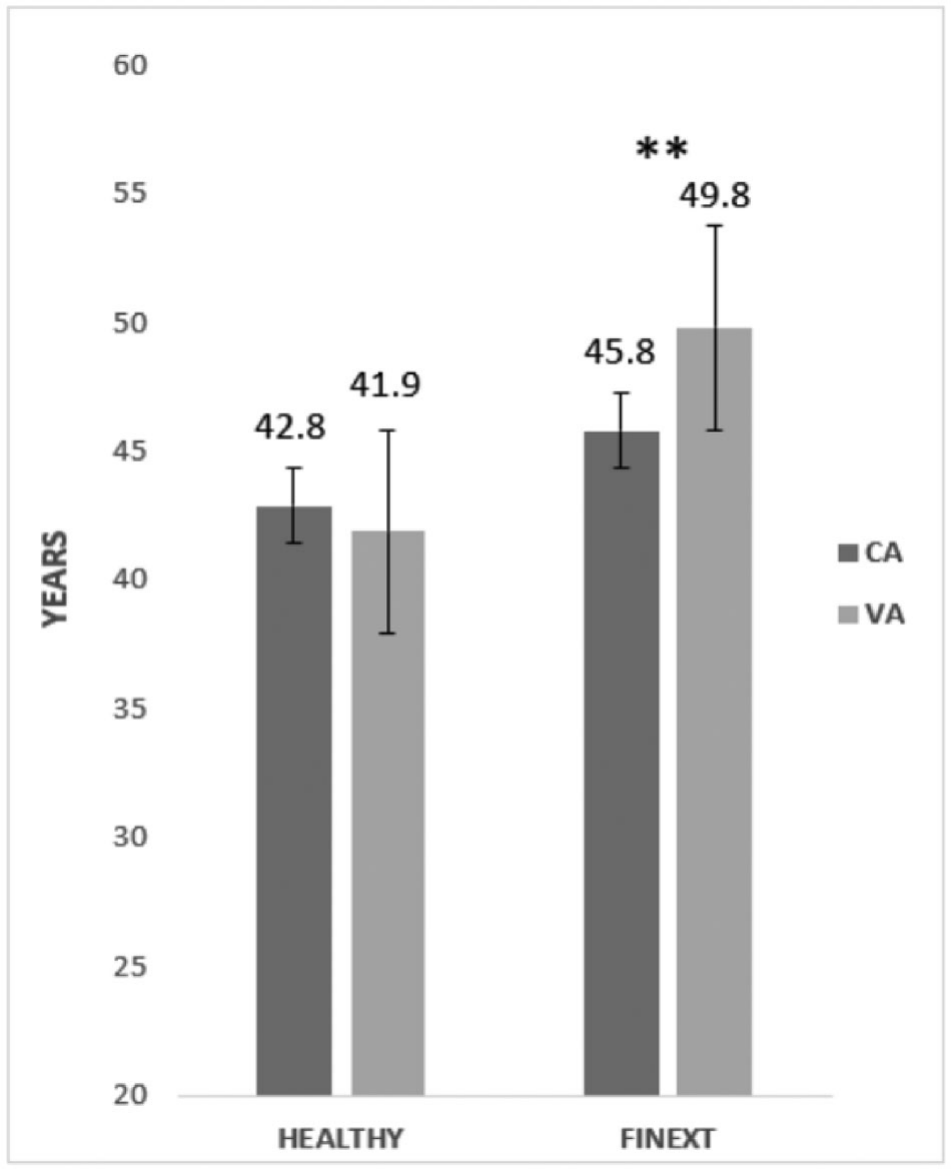
Comparison of vascular age (VA) and chronological age (CA) in the studied groups. ***p < 0.001; **p < 0.01; *p < 0.05: significant difference between FINEXT‐BD and healthy (HC).

The characteristics of CVR factors classified by the studied population are presented in Table 1. The absolute CVR differed significantly (*p* < 0.05) between groups, with HCs exhibiting a lower CVR than FINEXTs according to the SCORE2 method, but not the FHS method (SCORE2: mean difference = 0.955, 95% CI = 0.1393%−1.771%, *p* = 0.022; FHS: mean difference = 1.559, 95% CI = 1.254%−4.371%, *p* = 0556, Figure 1). Following SCORE2 risk assessment, FINEXT participants were considered high risk (3.1%) and HCs low‐to‐moderate risk (2.2%). However, using the FHS score, although with no statistical differences, the FINEXT group was considered medium risk (6.8%), whereas the HC group was regarded as low risk (5.2%). Furthermore, there were no signficant differences (*p* = 0.369) between groups according to relative risk (FINEXTs = 1.7%, HCs = 1.4%), with both groups considered low‐to‐moderate risk. The assessment of VA (Figure 2) showed significantly higher values in the FINEXTs than HCs (*p* = 0.012, mean difference = 7.928, 95% CI = 1.799–14.508 years old), with higher values of VA than chronological age in the FINEXT‐BD group (*p* = 0.005, mean difference = −4.048, 95% CI = −6.850 to −1.246 years old).

## 4 Discussion

For the first time, the present study measured physical, physiological, and biochemical health status markers of people with BD (i.e., the FINEXT sample) and provided an assessment of the physiopathology of the studied population compared to an HC group. Overall, BD participants showed unfavorable body composition, biochemical, CRF, and CVR profiles compared to those values in the HC group. Consequently, the BD population tended to have a higher CVR than HCs following SCORE2 and VA (according to the FHS) measures. These findings highlight the importance of CVR and VA assessment of people with BD to prevent CVD in clinical practice.

Previous studies have suggested that people with BD are more likely to have higher levels of abdominal obesity, lower rates of fat oxidation, and higher blood pressure than the general population (Fleet‐Michaliszym et al. 2008; Kang et al. 2024). In the present study, people with BD showed higher values in BMI (17.9%, *p* < 0.001, Cohen's *d* = 0.9), WHR (11.1%, *p* < 0.001, Cohen's *d* = 1.16), and FBM (37.03%, *p* < 0.001, Cohen's *d* = 0.94) than HCs. Body mass, specifically FBM gain, is a significant concern for people with BD (Torrent et al. 2008). Usually, in BD, long‐term treatment consists of mood stabilizers, such as lithium (100% of our participants took lithium), valproate, lamotrigine, and antipsychotic agents (Nierenberg et al. 2023). These pharmacological treatments are associated with body mass and FBM gain, lower metabolic rate, and an increase in appetite secondary to an improvement of mood, which is associated with worse insulin sensitivity and lipid oxidation (Correll et al. 2015; Torrent et al. 2008).

Furthermore, the sedative effects of some of these medications can reduce physical activity, thereby decreasing energy expenditure (Torrent et al. 2008). However, not all the effects are related to treatments, and the effects of medication are unclear (Kessing et al. 2024). Thus, a higher BMI is seen in untreated patients since the first stages of the disorder and in untreated patients (Liu et al. 2023). In any case, there is an interplay between BD patients’ medication use and lifestyle factors that could induce metabolic imbalances (Kang et al. 2024), with promotion strategies and lifestyle changes that may be considered adjuvant treatments. Indeed, lifestyle was shown to interact with higher physical activity and BMI associated with lower lithium levels (Zorrilla et al. 2023). A recent study showed two critical conclusions: First, a thinner brain cortical thickness was associated with BD itself, higher BMI, and treatment with antipsychotics; conversely, lithium treatment was related to greater cortical thickness (McWhinney et al. 2024). Likewise, the effects of lithium on symptomatology and comorbidity in BD are partially modulated by shared genetic factors, resulting in more beneficial effects for those with BD‐I than BD‐II. Thus, the need for more personalized treatment strategies is clear (Herrera‐Rivero et al. 2023).

Even though a recent umbrella review of systematic reviews and a meta‐analysis showed that BD was significantly associated with primary hypertension (Kang et al. 2024) and that overweight and obesity are commonly associated with hypertension, the sample of the present study showed optimal blood pressure values (118/80 mmHg), with no significant difference compared to HCs (124/79 mmHg). Our sample was residing in a European country, and despite the sample being overweight (29.1 ± 6.1 kg m^2^), our results cannot be compared with data from studies in North America. Furthermore, prolonged antipsychotic treatment (66% of the participants in the present study were undergoing treatment with a combination of lithium and low‐dose antipsychotics) has been associated with lower systolic blood pressure due to an α‐adrenergic blockade (Correll et al. 2015).

The intrinsic biology of BD may increase CVR since it is recognized as a multisystem illness involving systemic inflammation, and its interplay with dyslipidemia may lead to chronic vascular remodeling and atherosclerosis in arteries (Marshe et al. 2017; Reponen et al. 2020). Thus, participants from the current study showed higher and abnormal values of cigarette smoking (50% vs. 10%), atherogenic lipids ratios such as TG/HDL‐C ratio (4.1 ± 1.5 vs. 3.3 ± 1.0), TC/HDL‐C (2.8 ± 2.3 vs. 1.5 ± 1), and CRP (3.8 ± 10.2 vs. 0.9 ± 1.05) compared to HCs, respectively, which may be associated with worse psychiatric symptomatology. These elevated, causative, or correlational values are markers of increased CVR (high risk, 3.1%) compared to HCs (low‐to‐moderate risk, 2.2%) observed in our sample (Figure 1), according to SCORE2. Moreover, VA (estimated by FHS, Figure 2) was significantly higher (*p* < 0.01) than chronological age in the FINEXT sample (49.8 years vs. 45.8 years), and no differences were found (*p* > 0.05) in the HCs (41.9 years vs. 42.8 years) (Figure 2). These results confirm those recently found with a large sample of people with BD, where CVR factors, as the leading causes of relative excess mortality, highlighted the contribution of structural and functional cardiac disorders to overall excess mortality along with coronary heart disease in BD (Paljärvi et al. 2024). Further, it is well known the bi‐directionality of CVD and psychiatric symptoms in mental illness in general, and in BD in particular, may exacerbate suicidal ideation (Biazus et al. 2023). Thus, suicidality is a complex phenomenon with cognitive, neurophysiological, environmental, and social causal factors at play. However, in BD, suicidality has a significant physiological component aggravated by symptomatic periods that may worsen as CVR factors also worsen (Carls‐Diamante and Atanasova 2025). Therefore, it would be helpful to analyze the interaction among CVR, CRF, psychiatric symptoms, and suicidal ideation to understand how the physiological conditions may contribute to cognition.

Thus, CVR and VA estimation predictions may represent a useful clinical tool to improve the quality of somatic treatments, as well as detecting patients at risk of a cardiac event, estimating the combined outcome of fatal and non‐fatal CVD events, communicating information about CVR, and understanding what the particular risk means in terms of life (SCORE2 Working Group and ESC Cardiovascular Risk Collaboration, 2021). Adding to that, results from the prediction and management of CVR in people with severe mental illnesses (PRIMROSE) have concluded that new models are including not only CVD risk factors but also data related to social deprivation, psychiatric diagnosis, prescription of antidepressant and antipsychotics, and alcohol report performed better on people with mental disorders than traditional risk score models (Osborn et al. 2015). However, for the present study, standardized tools, such as SCORE2, FHS, and VA, were used to compare the two populations (BD vs. HC) and avoid bias.

Recently, identifying the biological substrates that integrate the experiences and physiological processes underlying the observed psychological changes has become important. The mitochondrion represents a nexus of biological, psychological, and social factors with functions in energetics, cell signaling, and hormone production, suggesting a role for mitochondrial dysfunction in the pathological processes underlying psychiatric disorders (Daniels, Olsen, and Tyrka 2020). Skeletal muscle mitochondrial oxidative capacity is an integral contributor to CRF, along with the cardiopulmonary system's ability to supply oxygen to contracting muscles. In the present study, as a novelty, objectively measured parameters related to CRF were analyzed. The significantly lower levels of CRF observed in FINEXTs (−33.6%, *p* < 0.001, Cohen's *d* = 0.97) compared to HCs (V̇O_2peak_: 25 ± 8.2 vs. 33.4 ± 8.7 mL kg^−1^ min^−1^; MET_peak_: 7.1 ± 2.3 vs. 9.5 ± 2.4; VT_1_: 15.3 ± 4.8 vs. 25.2 ± 7.3 mL kg^−1^ min^−1^), combined with higher COP scores (i.e., less ventilatory efficiency) (27.9 ± 4.2 vs. 23.7 ± 4.2), respectively, may predict all‐cause mortality and CVD (Peterman et al. 2022). These results align with those previously found with a small sample of the BD population, confirming an association with a 20% increased premature mortality risk (Vancampfort et al. 2016). It has also been recently stated that people with serious mental disorders (including BD) showed a significant reduction in lung function with smoking and obesity patterns (Ruiz‐Rull et al. 2024). Several factors contributed to these differences, including negative symptoms, elevated depression levels, lack of access to facilities, socioeconomic deprivation, low self‐efficacy in physical activity, limited awareness of its benefits, and variations in mood, which can lead to decreased physical activity levels and consequently affect CRF (Faurholt‐Jepsen et al. 2023). Thus, enhancing mitochondrial function through exercise and CRF might be associated with considerable (10%–25%) improvements in survival and direct brain and mental health benefits. Indeed, a higher CRF has been associated with improved cognitive function, better mood regulation, and a possible reduction in the risk of neurodegenerative diseases (Heo, Noble, and Call 2023). In this sense, a previous study in adolescents with BD concluded that the lower the CRF, the greater the depressive symptoms, raising the question of whether a higher CRF could reduce the rate of depression in this young population (Popel et al. 2021). A pilot study with BD also found that CRF is associated with mental and physical health–related quality of life (Vancampfort et al. 2017).

Overall, the results of this preliminary analysis of a population with BD, presenting CVR factors, spotlight the need to promote transdisciplinary healthy lifestyle programs for all people, including those mentally ill, to prevent premature death from somatic causes. Implementing a supervised and individually designed adjuvant exercise program should be a priority to mitigate the impact of medication and/or illnesses on obesity and related health issues in individuals with BD.

## 5 Strengths and Limitations

The strengths of the present study are comparing two populations for better characterization and using gold‐standard CRF assessment. Nevertheless, there are some limitations. First, this was not a large epidemiological study; it was conducted at a single institution, and all patients were White, non‐Hispanics, which may limit the generalizability of our findings. Second, because the sample was not large, we did not differentiate between sexes, which could help to reveal differences between men and women. Last, the prognosis was not differentiated between BD‐I and BD‐II, which could have also revealed differences in the influence of medication.

## Author Contributions

**José Etxaniz‐Oses**: investigation, formal analysis, data curation, writing–original draft, visualization. **Sara Maldonado‐Martín**: supervision, writing–review and editing, methodology, investigation, conceptualization, validation, visualization, writing–original draft. **Inaki Zorrilla**: supervision, funding acquisition, investigation, resources, writing–review and editing. **Ilargi Gorostegi‐Anduaga**: methodology, data curation, supervision, validation, visualization, investigation, conceptualization, writing–review and editing. **Maria J. Apodaca‐Arrizabalaga**: resources, data curation, methodology, investigation, writing–review and editing. **Ana González‐Pinto**: project administration, writing–review and editing, methodology, funding acquisition, conceptualization, investigation, validation, visualization, resources, supervision.

## Ethics Statement

The local ethics committee and the University Hospital of Alava Ethics Committee approved the study (September 20, 2019, Certificate No. 2019‐036) following the Declaration of Helsinki II. This study is registered with the international standard for randomized controlled trials (NCT04400630).

## Conflicts of Interest

The authors declare no conflicts of interest. Dr Gonzalez‐Pinto has received grants and served as a consultant, advisor, or CME speaker for Janssen‐Cilag, Lundbeck, Otsuka, Alter, Angelini, Novartis, Rovi, Takeda, the Spanish Ministry of Science and Innovation (CIBERSAM), the Ministry of Science (Carlos III Institute), the Basque Government, and the European Framework Program of Research.

### Peer Review

The peer review history for this article is available at https://publons.com/publon/10.1002/brb3.70297.

## Data Availability

Data supporting this study are available under the request.

## References

[brb370297-bib-0001] American Psychiatric Association . 2013. Diagnostic and Statistical Manual of Mental Disorders *(5th Ed)* . 10.1176/appi.books.9780890425596.

[brb370297-bib-0002] Biazus, T. B. , G. H. Beraldi , L. Tokeshi , et al. 2023. “All‐Cause and Cause‐Specific Mortality Among People With Bipolar Disorder: A Large‐Scale Systematic Review and Meta‐Analysis.” Molecular Psychiatry 28, no. 6: 2508–2524. 10.1038/s41380-023-02109-9. 37491460 PMC10611575

[brb370297-bib-0003] Carls‐Diamante, S. , and N. Atanasova . 2025. “Psychoneural Reduction Revised: The Case of Suicidality in Bipolar Disorder.” European Journal of Neuroscience 61, no. 1: e16640. 10.1111/ejn.16640. 39723733 PMC11670439

[brb370297-bib-0004] Correll, C. U. , J. Detraux , J. De Lepeleire , and M. De Hert . 2015. “Effects of Antipsychotics, Antidepressants and Mood Stabilizers on Risk for Physical Diseases in People With Schizophrenia, Depression and Bipolar Disorder.” World Psychiatry 14, no. 2: 119–136. 10.1002/wps.20204. 26043321 PMC4471960

[brb370297-bib-0005] D´Agostino, R. B. , V. Sr , R. S. Pencina , et al. 2008. “General Cardiovascular Risk Profile for Use in Primary Care: The Framingham Heart Study.” Circulation 117, no. 6: 743–753. 10.1161/circulationaha.107.699579. 18212285

[brb370297-bib-0006] Daniels, T. E. , E. M. Olsen , and A. R. Tyrka . 2020. “Stress and Psychiatric Disorders: The Role of Mitochondria.” Annual Review of Clinical Psychology 16: 165–186. 10.1146/annurev-clinpsy-082719-104030. PMC8007172 32092280

[brb370297-bib-0007] De Hert, M. , J. M. Dekker , D. Wood , K. G. Kahl , R. I. G. Holt , and H.‐J. Möller . 2009. “Cardiovascular Disease and Diabetes in People With Severe Mental Illness Position Statement From the European Psychiatric Association (EPA), Supported by the European Association for the Study of Diabetes (EASD) and the European Society of Cardiology (ESC).” European Psychiatry 24, no. 6: 412–424. 10.1016/j.eurpsy.2009.01.005. 19682863

[brb370297-bib-0008] Faurholt‐Jepsen, M. , J. Busk , J. E. Bardram , et al. 2023. “Mood Instability and Activity/Energy Instability in Patients With Bipolar Disorder According to Day‐to‐Day Smartphone‐Based Data—An Exploratory Post Hoc Study.” Journal of Affective Disorders 334: 83–91. 10.1016/j.jad.2023.04.139. 37149047

[brb370297-bib-0009] Fleet‐Michaliszym, S. B. , I. Soreca , A. D. Otto , et al. 2008. “A Prospective Observational Study of Obesity, Body Composition, and Insulin Resistance in 18 Women with Bipolar Disorder and 17 Matched Control Subjects.” Journal of Clinical Psychiatry 69, no. 12: 1892–1900. 10.4088/JCP.v69n1207. 19026257 PMC3428955

[brb370297-bib-0010] García, S. , C. Bermadez‐Ampudia , S. Maldonado‐Marta‐n , et al. 2020. “Functionality and Neurocognition in Patients with Bipolar Disorder After a Physical‐Exercise Program (FINEXT‐BD Study): Protocol of a Randomized Interventionist Program.” Frontiers in Psychiatry 11: 568455. 10.3389/fpsyt.2020.568455. 33240125 PMC7670851

[brb370297-bib-0011] Goldstein, B. I. , B. T. Baune , D. J. Bond , et al. 2020. “Call to Action Regarding the Vascular‐Bipolar Link: A Report From the Vascular Task Force of the International Society for Bipolar Disorders.” Bipolar Disorders 22, no. 5: 440–460. 10.1111/bdi.12921. 32356562 PMC7522687

[brb370297-bib-0012] Goldstein, B. I. , M. R. Carnethon , K. A. Matthews , et al. 2015. “Major Depressive Disorder and Bipolar Disorder Predispose Youth to Accelerated Atherosclerosis and Early Cardiovascular Disease: A Scientific Statement from the American Heart Association.” Circulation 132, no. 10: 965–986. 10.1161/CIR.0000000000000229. 26260736

[brb370297-bib-0013] Harber, M. P. , L. A. Kaminsky , R. Arena , et al. 2017. “Impact of Cardiorespiratory Fitness on All‐Cause and Disease‐Specific Mortality: Advances Since 2009.” Progress in Cardiovascular Diseases 60, no. 1: 11–20. 10.1016/j.pcad.2017.03.001. 28286137

[brb370297-bib-0014] Heo, J. , E. E. Noble , and J. A. Call . 2023. “The Role of Exerkines on Brain Mitochondria: A Mini‐Review.” Journal of Applied Physiology 134, no. 1: 28–35. 10.1152/japplphysiol.00565.2022. 36417200 PMC9799148

[brb370297-bib-0015] Herrera‐Rivero, M. , K. Gutiérrez‐Fragoso , A. Thalamuthu , et al. 2023. “Immunogenetics of Lithium Response and Psychiatric Phenotypes in Patients With Bipolar Disorder.” Research Square 00: 00. 10.21203/rs.3.rs-3068352/v1. PMC10991481 38570518

[brb370297-bib-0016] Kaminsky, L. A. , R. Arena , Ø. Ellingsen , et al. 2019. “Cardiorespiratory Fitness and Cardiovascular Disease—The Past, Present, and Future.” Progress in Cardiovascular Diseases 62, no. 2: 86–93. 10.1016/j.pcad.2019.01.002. 30639135

[brb370297-bib-0017] Kang, J. , H. Lee , J. Park , et al. 2024. “Comorbid Physical Health Outcomes in Patients With Bipolar Disorder: An Umbrella Review of Systematic Reviews and Meta‐analyses.” Asian Journal of Psychiatry 99: 104138. 10.1016/j.ajp.2024.104138. 38991375

[brb370297-bib-0018] Kessing, L. V. , A. González‐Pinto , A. Fagiolini , et al. 2021. “DSM‐5 and ICD‐11 Criteria for Bipolar Disorder: Implications for the Prevalence of Bipolar Disorder and Validity of the Diagnosis—A Narrative Review From the ECNP Bipolar Disorders Network.” European Neuropsychopharmacology 47: 54–61. 10.1016/j.euroneuro.2021.01.097. 33541809

[brb370297-bib-0019] Kessing, L. V. , M. B. Knudsen , H. C. W. Rytgaard , C. Torp‐Pedersen , and M. Berk . 2024. “Lithium Versus Anticonvulsants and the Risk of Physical Disorders—Results From a Comprehensive Long‐Term Nation‐Wide Population‐Based Study Emulating a Target Trial.” European Neuropsychopharmacology 84: 48–56. 10.1016/j.euroneuro.2024.04.009. 38663126

[brb370297-bib-0020] Laukkanen, J. A. , S. K. Kunutsor , C. G. Araújo , and K. Savonen . 2021. “Cardiorespiratory Optimal Point During Exercise Testing Is Related to Cardiovascular and All‐Cause Mortality.” Scandinavian Journal of Medicine & Science in Sports 31, no. 10: 1949–1961. 10.1111/sms.14012. 34189765

[brb370297-bib-0021] Liu, Q. , L. Wang , F. Zhen , and C. An . 2023. “Occurrence of Metabolic Syndrome in Untreated Bipolar Disorders: A Cross‐Sectional Study.” Acta Neuropsychiatrica 16, 1–6. 10.1017/neu.2023.47. 37842830

[brb370297-bib-0022] Marshe, V. S. , S. Pira , O. Mantere , et al. 2017. “C‐Reactive Protein and Cardiovascular Risk in Bipolar Disorder Patients: A Systematic Review.” Progress in Neuro‐Psychopharmacology & Biological Psychiatry 79, no. Pt B: 442–451. 10.1016/j.pnpbp.2017.07.026. 28764912

[brb370297-bib-0023] McWhinney, S. R. , J. Hlinka , E. Bakstein , et al. 2024. “Principal Component Analysis as an Efficient Method for Capturing Multivariate Brain Signatures of Complex Disorders‐ENIGMA Study in People With Bipolar Disorders and Obesity.” Human Brain Mapping 45, no. 8: e26682. 10.1002/hbm.26682. 38825977 PMC11144951

[brb370297-bib-0024] Nierenberg, A. A. , B. Agustini , O. Köhler‐Forsberg , et al. 2023. “Diagnosis and Treatment of Bipolar Disorder: A Review.” JAMA 330, no. 14: 1370–1380. 10.1001/jama.2023.18588. 37815563

[brb370297-bib-0025] Osborn, D. P. J. , S. Hardoon , R. Z. Omar , et al. 2015. “Cardiovascular Risk Prediction Models for People With Severe Mental Illness: Results From the Prediction and Management of Cardiovascular Risk in People With Severe Mental Illnesses (PRIMROSE) Research Program.” JAMA Psychiatry 72, no. 2: 143–151. 10.1001/jamapsychiatry.2014.2133. 25536289 PMC4353842

[brb370297-bib-0026] Paljärvi, T. , K. Herttua , H. Taipale , et al. 2023. “Cause‐Specific Excess Mortality After First Diagnosis of Bipolar Disorder: Population‐Based Cohort Study.” BMJ Mental Health 26, no. 1: e300700. 10.1136/bmjment-300700. PMC10391789 37463759

[brb370297-bib-0027] Paljärvi, T. , K. Herttua , H. Taipale , M. Lähteenvuo , A. Tanskanen , and J. Tiihonen . 2024. “Cardiovascular Mortality in Bipolar Disorder: Population‐Based Cohort Study.” Acta Psychiatrica Scandinavica 150, no. 2: 56–64. 10.1111/acps.13715. 38826056

[brb370297-bib-0028] Peterman, J. E. , M. P. Harber , B. S. Fleenor , M. H. Whaley , C. G. Araújo , and L. A. Kaminsky . 2022. “Cardiorespiratory Optimal Point Is a Submaximal Exercise Test Variable and a Predictor of Mortality Risk.” Journal of Cardiopulmonary Rehabilitation and Prevention 42, no. 6: E90–E96. 10.1097/HCR.0000000000000711. 35861956 PMC9662820

[brb370297-bib-0029] Popel, N. , K. G. Kennedy , L. Fiksenbaum , R. H. B. Mitchell , B. J. MacIntosh , and B. I. Goldstein . 2021. “Clinical and Neuroimaging Correlates of Cardiorespiratory Fitness in Adolescents With Bipolar Disorder.” Bipolar Disorders 23, no. 3: 274–283. 10.1111/bdi.12993. 32960499

[brb370297-bib-0030] Radua, J. , L. Fortea , J. M. Goikolea , et al. 2023. “Meta‐Analysis of the Effects of Adjuvant Drugs in Co‐Occurring Bipolar and Substance Use Disorder.” Revista De Psiquiatria Y Salud Mental 00: 00. 10.1016/j.rpsm.2023.01.005. 37689524

[brb370297-bib-0031] Reponen, E. J. , I. Dieset , M. Tesli , et al. 2020. “Atherogenic Lipid Ratios Related to Myeloperoxidase and C‐Reactive Protein Levels in Psychotic Disorders.” Frontiers in Psychiatry 11: 672. 10.3389/fpsyt.2020.00672. 32754070 PMC7365890

[brb370297-bib-0032] Ruiz‐Rull, C. , M. J. Jaén‐Moreno , G. I. del Pozo , et al. 2024. “Low Lung Function in Bipolar Disorder and Schizophrenia: A Hidden Risk.” Frontiers in Physiology 15: 1335798. https://www.frontiersin.org/journals/physiology/articles/10.3389/fphys.2024.1335798. 38737830 10.3389/fphys.2024.1335798 PMC11084671

[brb370297-bib-0033] SCORE2 Working Group and ESC Cardiovascular Risk Collaboration . 2021. “SCORE2 Risk Prediction Algorithms: New Models to Estimate 10‐Year Risk of Cardiovascular Disease in Europe.” European Heart Journal 42, no. 25: 2439–2454. 10.1093/eurheartj/ehab309. 34120177 PMC8248998

[brb370297-bib-0034] Torrent, C. , B. Amann , J. Sánchez‐Moreno , et al. 2008. “Weight Gain in Bipolar Disorder: Pharmacological Treatment as a Contributing Factor.” Acta Psychiatrica Scandinavica 118, no. 1: 4–18. 10.1111/j.1600-0447.2008.01204.x. 18498432

[brb370297-bib-0035] Vancampfort, D. , N. Hagemann , S. Wyckaert , et al. 2017. “Higher Cardio‐Respiratory Fitness Is Associated With Increased Mental and Physical Quality of Life in People With Bipolar Disorder: A Controlled Pilot Study.” Psychiatry Research 256: 219–224. 10.1016/j.psychres.2017.06.066. 28646785

[brb370297-bib-0036] Vancampfort, D. , P. Sienaert , S. Wyckaert , et al. 2016. “Cardiorespiratory Fitness in Outpatients With Bipolar Disorder Versus Matched Controls: An Exploratory Study.” Journal of Affective Disorders 199: 1–5. 10.1016/j.jad.2016.03.057. 27046322

[brb370297-bib-0037] Zorrilla, I. , S. Lopez‐Zurbano , S. Alberich , et al. 2023. “Lithium Levels and Lifestyle in Patients With Bipolar Disorder: A New Tool for Self‐Management.” International Journal of Bipolar Disorders 11, no. 1: 11‐x. 10.1186/s40345-023-00291-x. 36929031 PMC10020397

